# Disrupting the OTUD4-USP7 deubiquitinase complex to suppress herpesvirus replication: a novel antiviral strategy

**DOI:** 10.1371/journal.ppat.1013052

**Published:** 2025-04-10

**Authors:** Shaowei Wang, Xuezhang Tian, Yunhong Zhong, Xiaoyu Xie, Ming Gao, Chuchu Zhang, Xi Cheng, Yining Qi, Bo Zhong, Pinghui Feng, Ke Lan, Junjie Zhang

**Affiliations:** 1 State Key Laboratory of Oral & Maxillofacial Reconstruction and Regeneration, Key Laboratory of Oral Biomedicine Ministry of Education, Hubei Key Laboratory of Stomatology, School & Hospital of Stomatology, State Key Laboratory of Virology and Biosafety, Medical Research Institute, Wuhan University, Wuhan, China; 2 Frontier Science Center for Immunology and Metabolism, Medical Research Institute, Wuhan University, Wuhan, China; 3 Hubei Key Laboratory of Tumor Biological Behavior, Zhongnan Hospital of Wuhan University, Wuhan, China; 4 Department of Colorectal and Anal Surgery, Clinical Center of Intestinal and Colorectal Diseases of Hubei Province, Zhongnan Hospital of Wuhan University, Wuhan, China; 5 Hubei Key Laboratory of Intestinal and Colorectal Diseases, Zhongnan Hospital of Wuhan University, Wuhan, China; 6 Department of Gastrointestinal Surgery, Medical Research Institute, Zhongnan Hospital of Wuhan University, Wuhan University, Wuhan, China; 7 Section of Infection and Immunity, Herman Ostrow School of Dentistry, Norris Comprehensive Cancer Center, University of Southern California, Los Angeles, California, United States of America; 8 State Key Laboratory of Virology, School of Life Sciences, Wuhan University, Wuhan, China; Wistar Institute, UNITED STATES OF AMERICA

## Abstract

The development of effective and broad-spectrum antiviral therapies remains an unmet need. Current virus-targeted antiviral strategies are often limited by narrow spectrum of activity and the rapid emergence of resistance. As a result, there is increasing interest in alternative approaches that target host cell factors critical for viral replication. One promising strategy is the targeting of deubiquitinases (DUBs), enzymes that regulate key host and viral proteins involved in viral reactivation and replication. In this study, we explore the potential of targeting a DUB complex for antiviral therapy based on our previous study. Our previous work revealed that the OTUD4-USP7 DUB complex plays a crucial role in KSHV lytic reactivation. Here, we developed a peptide, p8, which effectively disrupts the interaction between OTUD4 and USP7, leading to decreased abundance of the key viral transcription factor, RTA, and suppression of murine herpesvirus replication *in vivo*. These findings underscore the OTUD4-USP7 DUB complex as a promising host-targeting antiviral therapeutic target for the treatment of KSHV-associated malignancies. Moreover, our study highlights the potential of DUB-targeting therapies as a novel and effective strategy for the development of broad-spectrum antiviral agents.

## Author summary

Kaposi’s sarcoma-associated herpesvirus (KSHV) is an onco-herpesvirus associated with multiple human malignancies with no effective antiviral treatment available. Our previous study has revealed that the OTUD4-USP7 deubiquitinase complex plays a key role in stabilizing a critical KSHV transcription factor and promoting viral replication. In this study, we explored whether disrupting the OTUD4-USP7 interaction could serve as a novel antiviral strategy to inhibit KSHV reactivation. Traditional antiviral drugs typically target key viral components. Alternatively, targeting viral protein complexes or host factors crucial for viral replication has gained attraction. Despite these advancements, targeting host protein complexes for antiviral therapy remains largely underexplored. In this study, we employ both KSHV cell infection model and *in vivo* infection model of murine herpesvirus (MHV68) to demonstrate that disrupting the OTUD4-USP7 complex effectively suppresses the replication of both viruses. Our research provides proof-of-concept that targeting host protein-protein interactions (PPIs), specifically a deubiquitinase complex, can serve as a promising antiviral therapeutic strategy.

## Introduction

Viral pathogens pose significant health threat, causing a wide range of infectious diseases from pandemic outbreaks to chronic and latent infections. Prominent examples include the recent coronavirus disease 2019 (COVID-19) pandemic, while herpesvirus infections are highly prevalent and establish life-long infection, leading to diseases ranging from cold sores to cancer malignancies. The ongoing burden of these viral infections underscores the urgent need for novel and broad-spectrum antiviral therapies. Antiviral strategies are generally categorized into two approaches: virus-targeting and host-targeting [1]. Virus-targeting antivirals directly interfere with the viral machinery, primarily by inhibiting viral entry, viral enzymes, or other critical biological processes necessary for viral replication and assembly [2]. However, the narrow spectrum of activity and the rapid emergence of viral resistance present significant challenges to the effectiveness of virus-targeting therapies [3]. In contrast, host-targeting strategies aim to interfere with host factors critical for viral replication. These therapies exploit cellular components and metabolic pathways essential for viral replication, such as cellular kinases and proteases, nucleotide biosynthesis, and lipid metabolism [3,4]. While host-targeting strategies offer the potential for broad-spectrum antiviral effects, their clinical success has been limited thus far, primarily due to challenges such as toxicities and a lack of well-defined, druggable host targets. Therefore, identifying new host-targeting antiviral targets is critical for overcoming the limitations of current host-orientated antiviral therapies.

Herpesviruses are ubiquitous in the human population, causing a spectrum of diseases ranging from mild blisters to severe encephalitis and cancer. Herpesviruses are classified into three subfamilies–alpha-, beta-, and gamma-herpesviruses–based on different biological properties and tissue tropism [5]. Among these viruses, two gamma-herpesviruses, Kaposi’s sarcoma-associated herpesvirus (KSHV) and Epstein–Barr virus (EBV), constitute two of the seven well-recognized human oncoviruses, which together are causally linked to 15-20% of human cancers [6,7]. KSHV has been directly linked to several malignancies and inflammatory conditions, including Kaposi’s sarcoma (KS) [8], primary effusion lymphoma (PEL) and multicentric Castleman’s disease (MCD) [9,10], and inflammatory cytokine syndrome (KICS), a severe inflammatory condition [11–13]. Understanding the mechanisms of KSHV replication is critical for the development of novel therapeutic strategies. KSHV exhibits a biphasic life cycle, consisting of latent and lytic phases [14]. During latency, the virus persists in host cells with minimal gene expression, while the lytic phase involves viral reactivation, leading to the production of infectious virions [15]. This transition from latency to lytic replication, known as reactivation, is a critical aspect of the herpesvirus life cycle, particularly in the context of KSHV-associated malignancies [14,16].

In KSHV-associated cancers, such as Kaposi’s sarcoma (KS), tumor cells predominantly display latent viral infection, while spontaneous reactivation takes place in only 1–3% of infected cells [17]. However, accumulating evidence indicates that both latency and lytic infections contribute significantly to KS pathogenesis [17,18]. Despite the low percentage of lytic cells, lytic replication plays a critical role in KS pathogenesis [19–22]. Firstly, the production and release of infectious KSHV particles sustain the latently infected cell population by compensating for episomal loss during cell division and infected cell death [18]. Furthermore, lytic reactivation generates essential paracrine signals that promote tumorigenesis [23]. Strong clinical evidence supports this view. Ganciclovir, a nucleoside analogue that inhibits KSHV lytic replication, has demonstrated remarkable efficacy in randomized clinical trials for preventing KS progression [24–26]. Despite advances in treatment, patients with KSHV-related malignancies continue to face poor prognosis and high mortality rates. Therefore, extensive research has focused on understanding the mechanisms underlying viral reactivation and its contribution to viral pathogenesis. Central to the regulation of KSHV’s latent-lytic switch is the replication and transcription activator (RTA), encoded by the KSHV ORF50 gene. RTA functions as the master transcriptional regulator of lytic reactivation, initiating the expression of numerous early and late lytic genes [15]. Given the pivotal role of viral reactivation in oncogenesis, targeting this process represents an attractive strategy for therapeutic intervention against KSHV-associated malignancies. However, directly inhibiting transcriptional regulators like RTA remains highly challenging.

In our previous studies, we discovered that the deubiquitinase (DUB) complex OTUD4-USP7 plays a critical role in KSHV lytic replication [27]. Based on this discovery, we hypothesized that disrupting the OTUD4-USP7 interaction with a peptide inhibitor could destabilize RTA and inhibit KSHV reactivation. In this study, we developed a peptide, p8, which specifically disrupts the OTUD4-USP7 interaction, reducing RTA protein level and repressing gamma-herpesviruses replication. Further optimization of p8 led to the development of p8-DRI (D-retro-inverso), which efficiently suppressed the replication of a murine gamma-herpesvirus *in vivo*. These findings not only reveal the critical role of the OTUD4-USP7 complex in gamma-herpesviruses infection, but also highlight the broader potential of targeting host factors involved in viral replication.

## Results

### Targeting the OTUD4-USP7 complex represses KSHV lytic replication

**Fig 1:**
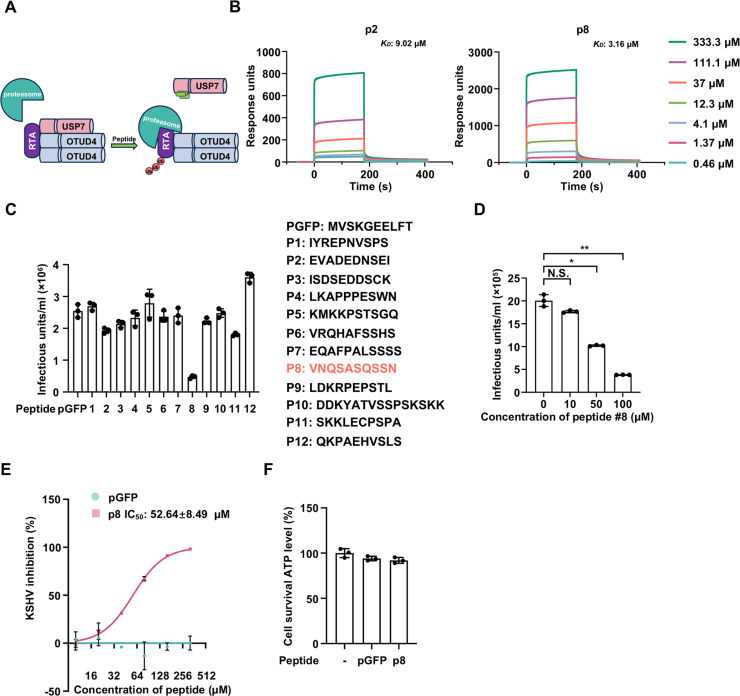
Rationally designed peptide p8 demonstrates anti-KSHV activity. (A) Graphical illustration showing the rationale underlying our peptide design. (B) Surface Plasmon Resonance (SPR) analysis assessing the interaction of the USP7-TRAF domain with the designed peptides p2 and p8. (C) SLK.iBAC-GFP cells were induced with doxycycline (Dox, 1 μg/mL) and sodium butyrate (0.5 mM) in the presence of the Tat fusion peptides (100 μM). All the peptides were fused to an N-terminal Tat_47-57_ peptide (YGRKKRRQRRR) via a flexible GG linker. The supernatants containing infectious virions were collected 48 h post-induction and used to infect HEK293T cells. KSHV infectious units were calculated based on the percentage of GFP-positive cells determined by flow cytometry. Data are mean ± s.d. of N = 3 independent biological replicates. (D) SLK.iBAC-GFP cells were induced with doxycycline (Dox, 1 μg/mL) and sodium butyrate (0.5 mM) in the presence of increasing amount of p8 (0, 10, 50, 100 μM). KSHV infectious units were determined 48 h post-induction. Data are mean ± s.d. of N = 3 independent biological replicates. (E) Comparative inhibitory activity of pGFP and p8 against KSHV lytic reactivation in SLK.iBAC-GFP cells. Data are mean ± s.d. of N = 3 independent biological replicates. (F) SLK.iBAC-GFP cells induced with Dox (1 μg/mL) and sodium butyrate (0.5 mM) were mock-treated or treated with pGFP or p8 (100 μM). Cell viability was assessed by measuring ATP levels 48 h post-induction. Data are mean ± s.d. of N = 3 independent biological replicates.

Given our findings that OTUD4 recruits USP7 to promote the deubiquitylation and stabilization of RTA, facilitating KSHV lytic replication [27], we hypothesized that disrupting the interaction between OTUD4 and USP7 might inhibit KSHV lytic replication. To test this hypothesis, we aimed to design a peptide to block the interaction between OTUD4 and USP7, thereby promoting RTA degradation to curb KSHV lytic replication. Our previous studies have revealed that the TRAF domain of USP7, rather than the catalytic domain (CD) or the tandem ubiquitin-like (UBL) domain, binds to the N-terminal region of OTUD4 [27]. Based on the consensus P/A/ExxS recognizing sequence of the USP7 TRAF domain [28], we synthesized a series of peptides derived from sequences within the N-terminal region of OTUD4 that may involve in USP7 binding. These peptides, designated p1 to p12 (Figs 1A and S1A), were analyzed for the ability to bind the USP7-TRAF domain. Surface plasmon resonance (SPR) analysis revealed that two peptides, p2 and p8, specifically bound to the USP7-TRAF domain, with dissociation constants (*K*_*D*_) of 9.02 μM and 3.16 μM, respectively (Fig 1B). To assess the antiviral activities of these peptides, we conjugated the peptides with the cell-penetrating peptide (CPP) Tat to facilitate cellular uptake [29]. Interestingly, we found that p8 effectively inhibited KSHV lytic reactivation in a dose-dependent manner, whereas the control pGFP peptide and p2 showed no significant inhibition (Figs 1C and 1D, and S1B and S1C Figs). Further analysis revealed that p8 inhibited KSHV lytic reactivation with an IC_50_ of 52.64 ± 8.49 μM, while IC_50_ for p2 could not be determined due to its low antiviral activity (Fig 1E and S1D Fig). Moreover, p8 treatment did not affect KSHV latent gene transcription or genome replication, indicating that p8 does not impact viral latency (S1E and S1F Fig). Additionally, we assessed the cytotoxicity of p8 and found that it exhibited minimal cytotoxicity on the cells (Fig 1F). Collectively, these data indicate that the rationally designed OTUD4-derived peptide p8, which binds to USP7, shows promising antiviral activity against KSHV lytic replication.

### P8 Disrupts the USP7-OTUD4 complex

**Fig 2:**
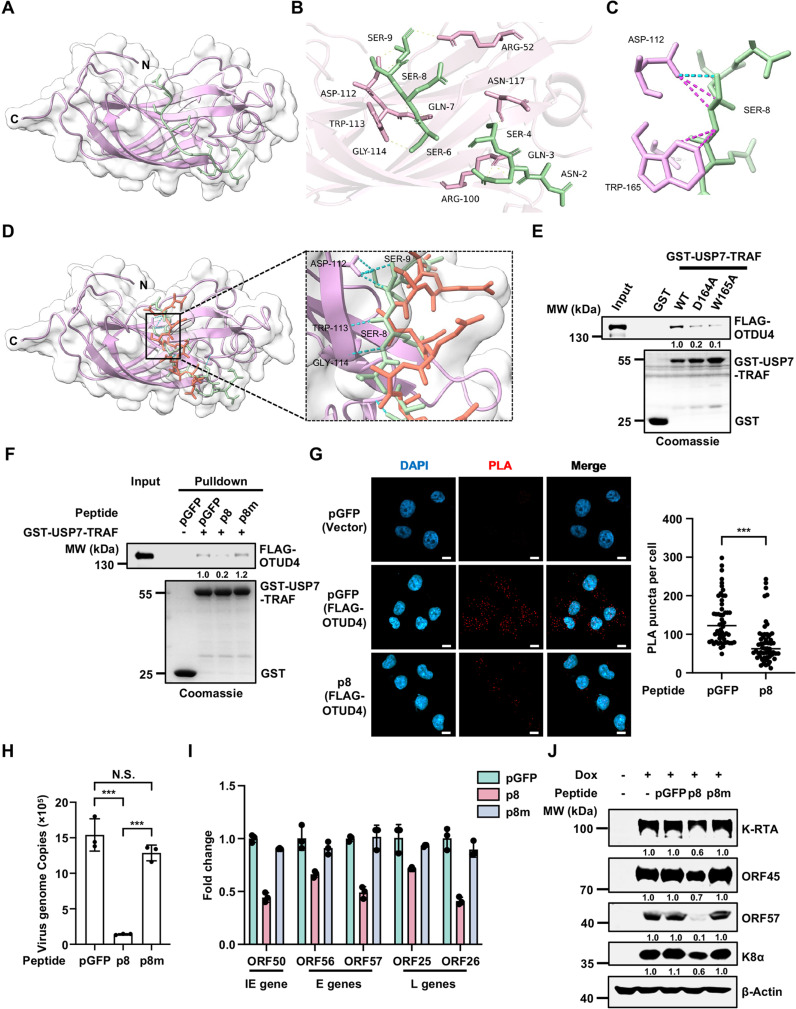
P8, but not the p8m mutant peptide, disrupts the USP7-OTUD4 complex and represses KSHV lytic reactivation. (A) Transparent surface representation of the USP7-TRAF domain (residues 53–208, plum) bound to peptide p8 (green) predicted by AlphaFold3. (B-C) Detailed interaction between USP7-TRAF (plum) and p8 (green) based on AlphaFold3. The H-bonds are indicated by dashed lines. (D) Structural overlay of USP7-TRAF (plum), p8 (green), and the p8m mutant peptide (orange) derived from AlphaFold3 prediction. The overall structure of USP7-TRAF bound to either p8 or p8m is shown in a transparent surface diagram (left), with detailed interaction sites highlighted on the right. The H-bonds are indicated by dashed lines. (E) Bacterially purified GST or GST fusion proteins (GST-USP7-TRAF WT, D164A or W165A) were incubated with FLAG-OTUD4 expressed in HEK293T cells for 4 h. Coomassie blue staining was used to visualize GST-tagged proteins, while western blotting was employed to detect FLAG-OTUD4. Densitometry analysis of the bands was performed using ImageJ. (F) Bacterially purified GST or GST-USP7-TRAF proteins were incubated with FLAG-OTUD4 expressed in HEK293T cells in the presence of the peptides [pGFP, p8, and p8m (100 μM)] for 4 h. Coomassie blue staining was used to visualize GST-tagged proteins, while western blotting was employed to detect FLAG-OTUD4. Densitometry analysis of the bands was performed using ImageJ. (G) FLAG-OTUD4-expressing SLK.iBAC-GFP stable cell lines were induced with Dox (1 μg/mL) and sodium butyrate (0.5 mM) to initiate lytic reactivation in the presence of pGFP or p8 peptides (100 μM). The cells were fixed and subjected to Proximity Ligation Assay (PLA) with FLAG and USP7 antibodies 24 h post-induction. Representative images (scale bars, 10 μm), and quantitative analysis of PLA puncta per cell were shown (Data are mean ± s.d. of N = 54 independent biological replicates for the pGFP group, and N = 58 independent biological replicates for the p8 group). (H-J) BCBL-1-Tet-K-RTA cells were induced with Dox (1 µg/mL) and sodium butyrate (0.5 mM) in the presence of the indicated peptides (100 µM). KSHV genome copies in the supernatants were quantified by qPCR 48 h post-induction (H), and viral gene expression and protein levels were analyzed by RT-qPCR (I) and immunoblotting (J). Data are mean ± s.d. of N = 3 independent biological replicates (H and I). Densitometry analysis of the bands was performed using ImageJ (J).

Our studies thus far suggest that p8 disrupts the USP7-OTUD4 complex to repress KSHV lytic replication. To elucidate the mechanism by which p8 interferes with the interaction between OTUD4 and USP7, we first utilized AlphaFold3 to predict the complex structure of the USP7-TRAF domain with p8 [30], given the availability of numerous crystal structures of the USP7-TRAF domain [31–36]. The overall structure of USP7-TRAF adopts a typical eight-stranded antiparallel β-sandwich configuration, forming a shallow groove on its surface (Fig 2A). The binding of p8 to USP7-TRAF is primarily mediated by a combination of hydrophilic and hydrophobic interactions with the β7 strand of USP7-TRAF, which contains a β-bulge structure (Fig 2B). Notably, nine hydrogen bonds are formed between p8 and the β7 strand of USP7-TRAF, five of which involve the Asp164 and Trp165 residues of USP7-TRAF (Fig 2B). Crucially, the key residue Ser8 of p8 forms hydrogen bonds and van der Waals interactions with Asp164 and Trp165 of USP7-TRAF, thereby dominating the binding interface (Fig 2C). Importantly, Ser8 confers a structural conformation on p8 that facilitates its optimal binding with USP7-TRAF, enabling the formation of a stable complex (Fig 2C). When Ser8 of p8 is mutated to Alanine (p8m), no interaction with USP7-TRAF is observed within 0.3 Å using AlphaFold3 prediction, in contrast to the strong interaction with the original p8 (Fig 2D).

To validate the structural predictions, we generated point mutations of the key residues in both USP7-TRAF and p8. Specifically, mutation of Asp164 or Trp165 in USP7-TRAF to Alanine significantly reduced OTUD4 binding in GST pull-down assays, confirming that Asp164 and Trp165 are critical for the interaction between OTUD4 and USP7-TRAF (Fig 2E). Moreover, while p8 effectively blocked the interaction between OTUD4 and USP7-TRAF, the p8m mutant (S8A) failed to do so (Fig 2F). Additionally, p2 failed to disrupt the interaction between USP7-TRAF and OTUD4 (S2A Fig). Next, we employed proximity ligation assay (PLA) to examine the impact of p8 on the interaction between OTUD4 and USP7 during viral infection. Since commercial OTUD4 antibodies failed to produce reliable immunofluorescence signals, we generated a FLAG-OTUD4 stable cell line. PLA results indicated that p8 treatment disrupted the co-localization of OTUD4 and USP7 (Fig 2G). Next, we selected several known substrates of USP7 (MDM2, p53 or ICP0) [37] to evaluate the specificity of p8. Our results indicate that p8 did not affect the interactions between USP7 and MDM2, p53, or ICP0 (S2B-S2D Figs), suggesting that p8 specifically disrupts the interaction between OTUD4 and USP7. These findings indicate that OTUD4 interacts with USP7 via the Asp164 and Trp165 residues of the USP7-TRAF domain, and that p8 disrupted the OTUD4-USP7 complex by specifically targeting the binding interface between OTUD4 and USP7.

### P8, but not the p8m mutant peptide, represses KSHV lytic reactivation

Our previous study revealed that KSHV hijacks the OTUD4-USP7 complex to facilitate lytic reactivation [27]. Therefore, we hypothesize that disrupting the interaction between OTUD4 and USP7 would repress KSHV lytic reactivation. Indeed, treatment with the p8 peptide, but not p8m, significantly reduced KSHV progeny virion production (S2E Fig), and decreased the transcription levels of multiple viral genes, including immediate-early gene ORF50, early genes ORF56 and ORF57, and late genes ORF25 and ORF26 (S2F Fig). Additionally, protein levels of RTA, ORF45, K8α, and ORF57 were also diminished following p8 treatment (S2G Fig). To further validate the function of p8 in repressing KSHV lytic reactivation, we utilized the BCBL-1-Tet-K-RTA cell line, a human primary effusion lymphoma (PEL) cell line that undergoes lytic reactivation upon doxycycline induction [38]. We confirmed that p8 significantly impaired KSHV lytic replication, demonstrated by reduced progeny viral genomic copy number, decreased transcription of KSHV lytic genes, and lower levels of viral proteins upon p8 treatment (Figs 2H-2J). These data collectively indicate that p8 represses KSHV lytic reactivation.

### P8 inhibits KSHV lytic reactivation by targeting the RTA-OTUD4-USP7 complex

**Fig 3:**
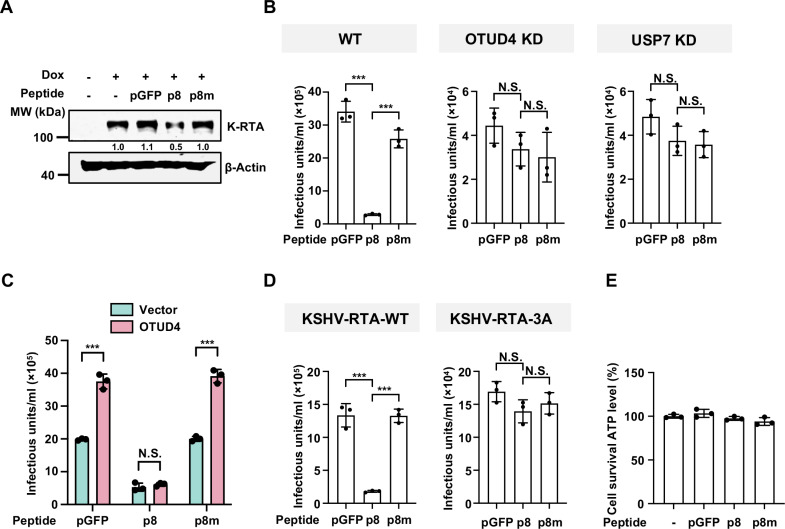
P8 inhibits KSHV lytic replication by targeting the RTA-OTUD4-USP7 complex. (A) iSLK cells were induced with Dox (1 µg/mL) in the presence of indicated peptide (100 µM). WCLs were analyzed by immunoblotting 48 h post-induction. Densitometry analysis of the bands was performed with ImageJ. (B) SLK.iBAC-GFP cells transduced with control shRNA, shRNA targeting OTUD4, or USP7 were induced with Dox (1 µg/mL) and sodium butyrate (0.5 mM) in the presence of the indicated peptide (100 µM). KSHV infectious units were quantified 48 h post-induction. Data are mean ± s.d. of N = 3 independent biological replicates. (C) SLK.iBAC-GFP cells stably transduced with vector control or OTUD4 were induced with Dox (1 µg/mL) and sodium butyrate (0.5 mM) in the presence of the indicated peptide (100 µM). KSHV infectious units were quantified 48 h post-induction. Data are mean ± s.d. of N = 3 independent biological replicates. (D) SLK.iBAC-K-RTA-WT or SLK.iBAC-K-RTA-F689A/R690A/D691A (3A) cells were induced with Dox (1 µg/mL) and sodium butyrate (0.5 mM) in the presence of the indicated peptide (100 µM). KSHV infectious units were quantified 48 h post-induction. Data are mean ± s.d. of N = 3 independent biological replicates. (E) SLK.iBAC-GFP cells were induced with Dox (1 µg/mL) and sodium butyrate (0.5 mM) in the presence of the indicated peptide (100 µM). Cell viability was assessed by measuring ATP levels 48 h post-induction. Data are mean ± s.d. of N = 3 independent biological replicates.

Given that p8 specifically disrupts the interaction between OTUD4 and USP7, and that the OTUD4-USP7 complex is critical for maintaining RTA stability [27], we hypothesized that p8 treatment promotes RTA degradation to repress KSHV lytic replication. Indeed, p8, but not the p8m mutant peptide, reduced the protein level of RTA in iSLK cells, a doxycycline-inducible K-RTA cell line [39] (Fig 3A). Additionally, while OTUD4 expression enhanced the protein level of RTA, p8, but not p8m, countered the promoting effect (S3A Fig). Consistently, p8, but not p8m, efficiently suppressed KSHV lytic reactivation. However, the antiviral effect of p8 was abolished in OTUD4-deficient or USP7-deficient cells (Figs 3B and S3B). Furthermore, OTUD4 expression promoted KSHV lytic replication, which can be negated by p8 but not p8m (Fig 3C). These data indicate that the antiviral activity of p8 against KSHV relies on the OTUD4-USP7 complex.

Previously, we identified the C-terminal FRD motif of RTA as crucial for recruiting the OTUD4-USP7 complex, and generated a KSHV-RTA-3A mutant by introducing F689A/R690A/D691A triple mutations into KSHV genome [27]. Since the RTA-3A mutant was unable to recruit OTUD4-USP7 to stabilize RTA, KSHV-RTA-3A demonstrates severely impaired lytic reactivation [27]. Remarkably, unlike KSHV-RTA-WT, p8 failed to suppress lytic reactivation of KSHV-RTA-3A (Fig 3D). Additionally, we assessed the cytotoxicity of p8 and p8m and found that they exhibited minimal cytotoxicity on the cells (Fig 3E). These data indicate that p8 reduces RTA level by disrupting the interaction between OTUD4 and USP7, thereby repressing KSHV lytic reactivation.

### OTUD4 deficiency impairs MHV68 replication

Previously, we discovered that OTUD4 plays a critical role in KSHV lytic reactivation [27]. Next, we asked whether OTUD4 also promotes the replication of MHV68, a murine herpesvirus that is genetically and biologically relevant to KSHV. Knockdown of OTUD4 in NIH3T3 cells significantly impaired MHV68 lytic replication, as indicated by the decreased GFP intensity (S4A and S4B Fig). Consistently, the infectious progeny virus titers were dramatically reduced in the OTUD4-depleted cells (S4C Fig). These data indicate that OTUD4 promotes MHV68 replication.

**Fig 4:**
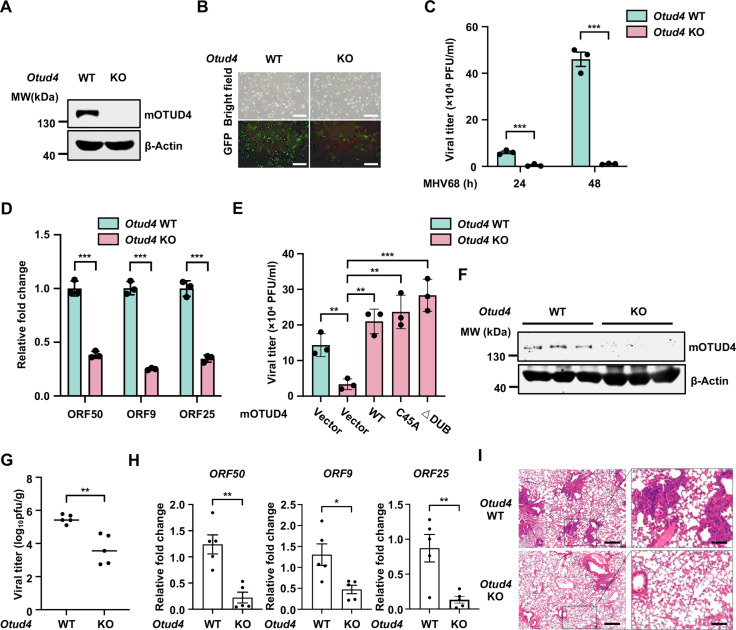
OTUD4 deficiency impairs MHV68 replication *in vivo.* (A) MLFs derived from *Otud4*^^*fl/fl*^^ Cre-ERT2^-/-^ and *Otud4*^^*fl/fl*^^ Cre-ERT2^+/-^mice were treated with 4-hydroxytamoxifen (1 μM) for 3 days to generate OTUD4 knockout cells. Whole-cell lysates (WCLs) were prepared and analyzed by immunoblotting. (B) MLF cells described in Fig 4A were infected with MHV68-GFP (MOI, 0.01). The supernatants containing infectious virions were collected 48 h post-infection and used to infect BHK21 cells. GFP expression was imaged 48 h post-infection. Scale bars, 100 μm. (C) OTUD4 knockout MLFs described in Fig 4A were infected with MHV68 (MOI, 0.01), and viral titer was determined at the indicated time points post-infection. Data are mean ± s.d. of N = 3 independent biological replicates. (D) OTUD4 knockout MLFs described in Fig 4A were infected with MHV68 (MOI, 0.01), and viral gene expression was quantified by RT-qPCR 48 h post-infection. Data are mean ± s.d. of N = 3 independent biological replicates. (E) OTUD4 knockout MLFs reconstituted with control vector, OTUD4, OTUD4-C45A, or OTUD4-ΔDUB were infected with MHV68-GFP (MOI, 0.01). MHV68 viral titer was quantified 48 h post-infection. Data are mean ± s.d. of N = 3 independent biological replicates. (F) *Otud4*^^*fl/fl*^^ Cre-ERT2^-/-^ and *Otud4*^^*f*^^*l/fl*^^ Cre-ERT2^+/-^ mice were intraperitoneally injected with tamoxifen for five consecutive days. The protein levels of OTUD4 in the lungs were analyzed by immunoblotting. (G-I) *Otud4*^^*fl/fl*^^ Cre-ERT2^-/-^ and *Otud4*^^*f*^^*l/fl*^^ Cre-ERT2^+/-^ mice described in Fig 4F were intranasally infected with MHV68 (4000 PFU). Viral gene expression (G) and viral titer in the lungs (H) were quantified 5 days post-infection, and mouse lung tissues were processed with H&E staining (I). Scale bars, 500 μm (left) and 100 μm (right). Data are mean ± s.d. of N = 5 independent biological replicates (G and H).

To explore whether OTUD4 has a critical role in herpesvirus replication *in vivo*, we turned to MHV68 for *in vivo* infection studies in mice, since KSHV exhibits strict host tropism and primarily infects humans as the only natural host [40–42]. MHV68 readily infects laboratory mice, leading to acute replication lasting around two weeks, and then establishes latency primarily within B-lymphocytes, providing a useful model for studying herpesvirus replication *in vivo* [43]. We generated *Otud4*^fl/fl^ mice and crossed them with ROSA26-CreERT2 mice to obtain *Otud4*^fl/fl^ Cre-ERT2^+/-^ and *Otud4*^fl/fl^ Cre-ERT2^-/-^ mice. Then, we generated primary mouse lung fibroblasts (MLFs) from these mice, and induced OTUD4 knockout with 4-hydroxytamoxifen and then performed MHV68 infection *ex vivo* (Fig 4A). Consistent with our previous findings [27], MHV68 replication was significantly reduced in OTUD4 knockout MLFs compared to control cells, as indicated by reduced GFP intensity and progeny virus production (Fig 4B and 4C). Additionally, the transcription levels of the viral genes (ORF50, ORF9, and ORF25) were greatly decreased in OTUD4-depleted cells (Fig 4D). To further confirm the role of OTUD4 in MHV68 replication, we performed rescue experiments. We reconstituted OTUD4-deficient MLF cells with an OTUD4 construct, a DUB-deficient C45A mutant, or a DUB deletion mutant (ΔDUB) (S4D Fig). While OTUD4 knockout markedly reduced MHV68 progeny viral titer, reconstitution of OTUD4 reversed the effect of OTUD4 deficiency on MHV68 replication (Fig 4E). Notably, the DUB-deficient mutants (C45A and ΔDUB) also effectively rescued MHV68 replication (Fig 4E), indicating that OTUD4 promotes MHV68 lytic replication independently of its DUB activity.

To investigate OTUD4’s role in MHV68 replication *in vivo*, we administered tamoxifen to *Otud4*^fl/fl^ Cre-ERT2^+/-^ and *Otud4*^fl/fl^ Cre-ERT2^-/-^ mice to induce OTUD4 knockout, followed by MHV68 infection via intranasal inoculation. As expected, tamoxifen treatment depleted OTUD4 in the lungs of *Otud4*^fl/fl^ Cre-ERT2^+/-^ mice but not in *Otud4*^fl/fl^ Cre-ERT2^-/-^ mice (Fig 4F). OTUD4 deficiency significantly reduced MHV68 viral titers in lung tissues (Fig 4G). Consistently, the transcription levels of multiple viral genes (ORF50, ORF9, and ORF25) were markedly decreased in the lungs of OTUD4-deficient mice compared to control mice (Fig 4H). Additionally, OTUD4-deficient mice exhibited less inflammation and immune cell infiltration in the lungs after MHV68 infection compared to control mice (Fig 4I). Collectively, these data indicate that OTUD4 deficiency impairs MHV68 lytic replication *in vivo*.

Our previous study revealed that OTUD4 promotes the deubiquitination of RTA protein to enhance its stability, thereby facilitating KSHV lytic reactivation [27]. Given that MHV68 is genetically and biologically closely related to KSHV, we hypothesized that OTUD4 might promote MHV68 lytic replication through a similar mechanism. Indeed, ectopic expression of wild type (WT) mOTUD4 (murine OTUD4), the C45A DUB-deficient mutant, or the ΔDUB mutant enhanced m-RTA (MHV68-RTA) protein levels (S4E Fig), consistent with our observations that the DUB activity of OTUD4 is not required for promoting MHV68 lytic replication (Fig 4E). Furthermore, human OTUD4 WT or the C45A mutant also increased m-RTA expression in a dose-dependent manner (S4F Fig), suggesting that the stabilization of RTA by OTUD4 is a conserved strategy among different species. Additionally, mOTUD4 WT, as well as the C45A and ΔDUB mutants, promoted the deubiquitination of m-RTA (S4G Fig). These data indicate that OTUD4 stabilizes m-RTA and promotes MHV68 replication, and suggest that the stabilization of RTA proteins by OTUD4 is a conserved strategy among gamma-herpesviruses.

### P8-DRI restricts MHV68 infection *in vivo*

**Fig 5:**
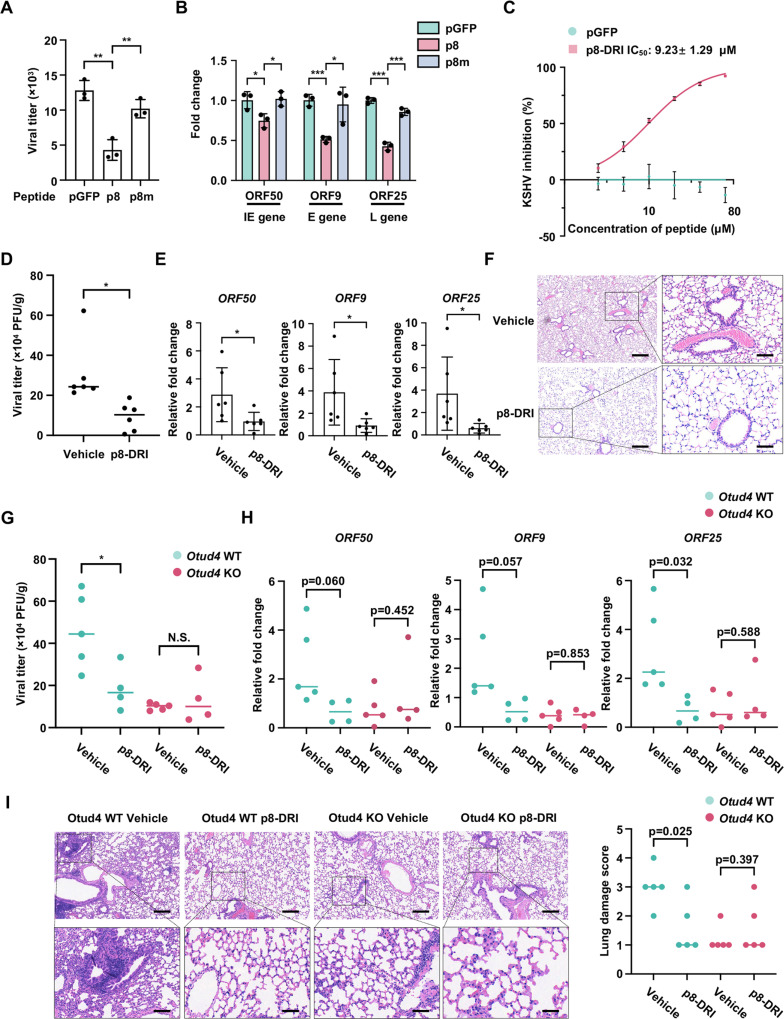
P8-DRI restricts MHV68 Infection *in vivo.* (A-B) MLF cells were infected with MHV68 (MOI, 0.01) and treated with the indicated peptides (100 µM). Viral titer was determined by plaque assays 48 h post-infection (A). Viral gene expression was assessed by RT-qPCR (B). Data are mean ± s.d. of N = 3 independent biological replicates. (C) Comparative inhibitory activity of pGFP and p8-DRI (nssqsasqnvpprrrqrrkkrGy) against KSHV lytic reactivation in SLK.iBAC-GFP cells. Data are mean ± s.d. of N = 3 independent biological replicates. (D-E) C57BL/6 mice were intranasally inoculated with MHV68 (4000 PFU) and administrated with p8-DRI (5 mg/kg of body weight) or vehicle control (n = 6 per group). Viral loads in lung tissues were quantified at 5 days post-infection (D). Viral gene expression in lung tissues was measured by RT-qPCR at 5 days post-infection (E). Data are mean ± s.d. of N = 6 independent biological replicates. (F) H&E staining of lung tissue sections from MHV68-infected mice treated with p8-DRI or vehicle 5 days post-infection. Scale bars, 500 µm (left) and 100 µm (right). (G-H) *Otud4*^^*fl/fl*^^ Cre-ERT2^-/-^ and *Otud4*^^*fl/fl*^^ Cre-ERT2^+/-^ mice were administered with tamoxifen via intraperitoneal injection. After 5 days, the mice were intranasally infected with of MHV68 (4000 PFU) and treated with p8-DRI (5 mg/kg of body weight, n = 4) or vehicle control (n = 5). Viral titers in lung tissues were determined at 5 days post-infection (G). Viral gene expression in lung tissues was assessed by RT-qPCR (H). Data are mean ± s.d. of N = 5 independent biological replicates for the vehicle group, and N = 4 independent biological replicates for the p8-DRI group. (I) H&E staining of lung tissue sections from MHV68-infected *Otud4*^^*fl/fl*^^ Cre-ERT2^-/-^ and *Otud4*^^*fl/fl*^^ Cre-ERT2^+/-^ mice treated with p8-DRI or vehicle control, analyzed at 5 days post-infection. Scale bars, 500 µm (left) and 100 µm (right). Histological evaluation and scoring of the lung tissues were performed by a blinded pathologist. Data are mean ± s.d. of N = 5 independent biological replicates.

Given that OTUD4 has a critical role in MHV68 replication and that MHV68 is genetically and biologically related to KSHV, we next assessed the antiviral activity of p8 against MHV68 *in vivo*. First, we performed sequence alignment of human and murine USP7 and OTUD4. USP7 is evolutionarily conserved, and sequence alignment of human and murine USP7-TRAF domains revealed >95% identity. Sequence alignment of the N-terminal regions of human and murine OTUD4, which is responsible for USP7-TRAF binding, also revealed high similarity (>85%) (S5A Fig). However, there are three amino acid differences between p8-m (murine-derived p8) and p8 (human-derived p8). To evaluate the binding, using AlphaFold3, we predicted the structure of the p8-murine USP7-TRAF complex. The binding mode exhibited structural congruence with human USP7-TRAF, suggesting that p8 can target both human and murine USP7-TRAF (S5B Fig). Similarly, AlphaFold3 structural prediction of the p8-m-murine USP7-TRAF complex revealed that binding is partly mediated by interactions between the N8 residue of p8-m and Asp165 and Trp166 of murine USP7-TRAF. Additionally, the Gln7 and Ser9 residues of p8-m also contributes to the interaction (S5C Fig). However, unlike p8, the N-terminus of p8-m is partially solvent-exposed in the complex. Subsequent mutagenesis experiments indicated that alanine substitution of Asp165 and Trp166 in murine USP7-TRAF greatly impaired its interaction with mOTUD4, suggesting that the USP7-OTUD4 binding interface near the p8 region is conserved between human and murine (S5D Fig). In line with the structural predictions, both p8 and p8-m effectively disrupted the interaction between murine USP7-TRAF and murine OTUD4, while p8 showed slightly stronger inhibitory activity (S5E Fig). More importantly, we found that p8 demonstrated potent antiviral activity against MHV68 in both MLF and NIH3T3 cells, as evidenced by a significant reduction in viral gene expression. In contrast, a control peptide pGFP and the p8m mutant peptide had no inhibitory effects (Figs 5A, 5B, and S5F and S5G Figs). These data indicate that p8 also effectively represses MHV68 replication.

Next, to improve the antiviral efficacy of p8, we modified it by replacing L-isomers with D-isomers and reversing the peptide sequence [44]. These DRI modifications are expected to improve peptide stability and antiviral potency, and have demonstrated excellent tolerance and therapeutic effectiveness [45–48]. As anticipated, the DRI-modified version of p8, referred to as p8-DRI, exhibited enhanced anti-KSHV activity, with an IC_50_ of 9.23 ± 1.29 μM (Fig 5C), significantly lower than the IC_50_ of the original p8 (52.64 ± 8.49 μM). Importantly, the binding affinity of p8-DRI for USP7-TRAF remained comparable to that of p8 (S5H Fig). In addition, our results revealed that the IC_50_ for p2-DRI was 67.41 ± 34.09 μM, which correlates with its low antiviral activity (S5I Fig).

To evaluate the antiviral efficacy of p8-DRI *in vivo*, we treated C57BL/6 mice with p8-DRI following intranasal infection with MHV68. Mice treated with p8-DRI showed lower viral titers in lung tissues compared to vehicle-treated controls (Fig 5D). Additionally, the transcription levels of multiple MHV68 viral genes (ORF50, ORF9, and ORF25) were significantly reduced in the lungs of p8-DRI-treated mice (Fig 5E). Moreover, p8-DRI-treated mice exhibited less inflammation and immune cell infiltration in the lungs after MHV68 infection compared to vehicle-treated control mice (Fig 5F). These data indicate that p8-DRI restricts MHV68 Infection *in vivo*. To investigate whether the *in vivo* antiviral activity of p8-DRI is dependent on OTUD4, we administered tamoxifen to *Otud4*^fl/fl^ Cre-ERT2^+/-^ and *Otud4*^fl/fl^ Cre-ERT2^-/-^ mice to induce OTUD4 knockout, followed by MHV68 infection and p8-DRI treatment. As expected, *Otud4*^fl/fl^ Cre-ERT2^-/-^ (OTUD4-replete) mice treated with p8-DRI showed lower viral titers in lung tissues and reduced viral pathology compared to vehicle-treated controls. By contrast, the antiviral effect of p8-DRI was abolished in OTUD4-deficient mice (Figs 5G-5I). These data indicate that p8-DRI targets OTUD4 to restrict MHV68 infection *in vivo*.

## Discussion

Our previous study has revealed that the OTUD4-USP7 deubiquitinase complex plays a critical role in KSHV reactivation. Specifically, OTUD4 functions as an adaptor protein that recruits USP7 to deubiquitinate and stabilize RTA, thereby promoting KSHV lytic reactivation [27]. In this study, we further explored the potential of targeting the OTUD4-USP7 deubiquitinase complex as a potential therapeutic strategy to inhibit viral replication. Our data indicate that knockout of OTUD4 in mice greatly impairs the replication of MHV68, a murine herpesvirus closely related to KSHV, thereby validating the critical role of OTUD4 in MHV68 replication *in vivo*. Through screening OTUD4-derived peptides, we identified a peptide, p8, which effectively blocks the interaction between OTUD4 and USP7 and suppresses the replication of both KSHV and MHV68. Importantly, our experimental evidence with genetically manipulated cells, viruses, and mouse infection models, indicates that p8 specifically targets the RTA-OTUD4-USP7 axis to exert antiviral activity.

Viral pathogens pose significant threats to human health, and antiviral therapies are vital for controlling viral replication and treating viral diseases. Traditional antiviral drugs, which typically target critical viral components to restrict viral replication, have achieved remarkable success [2,49]. However, viruses can rapidly develop resistance to these direct-acting antiviral agents by generating mutations. Therefore, there is growing interest in targeting viral protein complexes or host factors essential for viral replication [4,50]. Accumulating evidence indicates that host deubiquitinases play a critical role in viral infections. Therefore, deubiquitinases are exploited as therapeutic targets for antiviral therapies [51]. For example, silencing USP18 has been shown to promote the antiviral innate immunity against hepatitis C virus infection [52], while pharmacological inhibition of USP14 greatly suppresses murine norovirus infection [53]. Despite these advancements, targeting host deubiquitinase complexes for antiviral therapy has received less attention. Our study provides proof-of-principle that disrupting the OTUD4-USP7 complex effectively suppresses the replication of both KSHV and MHV68. This study illustrates the potential of targeting host protein-protein interactions (PPIs) critical for viral infection as a viable antiviral therapeutic strategy.

KSHV is the etiological agent of Kaposi’s sarcoma (KS) and primary effusion lymphoma (PEL), and contributes to other malignancies such as multicentric Castleman’s disease (MCD) [8–10]. Notably, the reactivation process has been recognized as essential for viral tumorigenesis [16,54]. Therefore, KSHV antiviral treatments, including those aimed at inhibiting lytic replication, are actively being developed [55]. For example, Nelfinavir prevents mature virion production by disrupting viral capsid assembly [56]. MMV1645152, identified from the Medicines for Malaria Venture (MMV) Pandemic Response Box, suppresses immediate-early and late lytic gene expression, effectively blocking KSHV lytic replication [57]. Additionally, selective inhibition of the ATPase activity of hTREX complex protein UAP56 by a small-molecule inhibitor CCT018159 successfully blocks KSHV lytic replication and infectious virion production [58]. Antagonists targeting various histamine receptors also strongly inhibit KSHV lytic replication [59]. Here, we present a prominent example in which a designed peptide specifically disrupts the interaction between OTUD4 and USP7 to exert potent antiviral activity. Further synthesis and development of peptidomimetic compounds based on peptide p8 could further improve antiviral efficacy and reduce costs [60].

In summary, we have developed an antiviral peptide inhibitor, p8, which effectively disrupts the interaction between OTUD4 and USP7, leading to decreased RTA levels and suppressing KSHV replication. Further optimization yielded p8-DRI, which exhibited enhanced anti-gamma-herpesvirus activity in both cellular and animal models. Our findings underscore the pivotal role of the OTUD4-USP7 DUB complex in gamma-herpesvirus infection and suggest that targeting this complex could serve as a potential antiviral therapeutic target for treating KSHV-associated malignancies. Moreover, our study highlights the potential of DUB-targeting therapies as a novel and effective strategy for the development of broad-spectrum antiviral agents.

## Materials and methods

### Ethics statement

All animal experiments were conducted in accordance with the guidelines approved by the Animal Care and Ethics Committee of Medical Research Institute, Wuhan University.

### Mice

*Otud4*^*fl/fl*^ and ROSA26-CreERT2 mice were kindly provided by Dr. Bo Zhong (Wuhan University) [61,62]. *Otud4*^*fl/fl*^ mice were crossed with ROSA26-CreERT2 mice to obtain *Otud4*^*fl/fl*^ Cre-ERT2^-/-^ and *Otud4*^*fl/fl*^ Cre-ERT2^+/-^ offspring.

To knock out OTUD4 in primary mouse lung fibroblasts (MLFs) derived from *Otud4*^*fl/fl*^ Cre-ERT2^-/-^ and *Otud4*^*fl/fl*^ Cre-ERT2^+/-^ mice, MLFs were treated with 4-hydroxytamoxifen (1 μM; Sigma, H6278) for 3 days. For *in vivo* depletion of OTUD4, 6- to 8-week-old *Otud4*^*fl/fl*^ Cre-ERT2^-/-^ and *Otud4*^*fl/fl*^ Cre-ERT2^+/-^ mice were injected intraperitoneally with tamoxifen (80 mg/kg body weight, dissolved in corn oil; MCE, HY-13757A) for 5 consecutive days. After another 5 days, the mice were inoculated intranasally with MHV68 (4000 PFU). The lungs of the infected mice were collected 5 days post-infection, and viral gene expression and viral titer were quantified by reverse transcription quantitative PCR (RT-qPCR) and plaque assays.

### Cell culture

HEK293T, NIH3T3 (ATCC), SLK, and BHK21 cells (kindly provided by Hongyu Deng, Institute of Biophysics, Chinese Academy of Sciences) were cultured in Dulbecco’s Modified Eagle Medium (DMEM) (Sigma) supplemented with 10% fetal calf serum (FCS) (Lonsera, Shuangra Biotech, Shanghai, China) and 1% penicillin-streptomycin (HyClone) [20,63]. iSLK cells carrying doxycycline-inducible K-RTA [39] (kindly provided by Drs. Jae Jung, Cleveland Clinic, and Kevin Brulois, Stanford University) were maintained in DMEM supplemented with 10% FCS, 1% penicillin-streptomycin, puromycin (1 μg/mL), and G418 (250 μg/mL). SLK cells carrying the KSHV clone iBAC [64] (SLK.iBAC-GFP; kindly provided by Dr. Fanxiu Zhu, Florida State University) were maintained in DMEM supplemented with 10% FCS, 1% penicillin-streptomycin, and hygromycin B (500 μg/mL). BCBL-1 cells (kindly provided by Dr. Ke Lan, Wuhan University) were maintained in RPMI-1640 (HyClone) supplemented with 10% FCS and 1% penicillin-streptomycin. The BCBL-1-Tet-RTA cell line was generated by stably transducing BCBL-1 cells with a doxycycline-inducible RTA construct [38]. Primary mouse lung fibroblasts were generated as previously described [65,66]. Briefly, mouse lungs were minced and digested in calcium- and magnesium-free Hank’s Balanced Salt Solution containing 10 μg/ml type II collagenase and 20 μg/ml DNase I (Invitrogen) at 37 °C for 1 h. Cell suspension was centrifuged at 300 × g for 5 min, and the cells were then plated in culture medium (1:1 [v/v] DMEM/Ham’s F-12 supplemented with 10% FCS, penicillin–streptomycin, 15 mM HEPES, and 2 mM L-glutamine).

### Antibodies and other reagents

The following antibodies and reagents were used for immunoblotting and immunoprecipitation: Mouse anti-FLAG monoclonal antibody (1:10000; Dia-An Biotechnology, Wuhan, China, catalog no. 2064); Mouse anti-HA monoclonal antibody (1:3000; Dia-An Biotechnology, Wuhan, China, catalog no. 2063); Mouse anti-β-actin monoclonal antibody (1:5000; Dia-An Biotechnology, Wuhan, China, catalog no. 2060); Rabbit anti-K-RTA monoclonal antibody (1:1000, Mabnus biotechnology, Wuhan, China); Rabbit anti-K-ORF55 polyclonal antibody (1:1000, Mabnus biotechnology, Wuhan, China); Mouse anti-KSHV ORF45 monoclonal antibody (1:1000; Santa Cruz, sc-53883); Mouse anti-KSHV ORF57 monoclonal antibody (1:1000; Santa Cruz, sc-135746); Mouse anti-c-Myc monoclonal antibody (1:1000; Santa Cruz, sc-40); Mouse anti-KSHV K8.1A/B monoclonal antibody (1:1000; Santa Cruz, sc-65446); Mouse anti-K8α monoclonal antibody (1:1000; Santa Cruz, sc-69797); Rabbit anti-OTUD4 polyclonal antibody (1:1000; Proteintech, 25070-1-AP); Mouse anti-USP7 monoclonal antibody (1:500; Santa Cruz, sc-137008); Rabbit anti-FLAG monoclonal antibody (ABclonal, AE092); IRDye 800CW Goat anti-Rabbit and anti-Mouse secondary antibodies (1:10000; LI-COR); Goat anti-Rabbit and anti-Mouse HRP Conjugated secondary antibodies (1:10000; Bio-Rad); Rabbit IgG (Proteintech, 20010049); Anti-FLAG agarose beads (Dia-An Biotechnology, Wuhan, China); Protein A/G agarose (GE Healthcare); Glutathione-Sepharose beads (Smart-lifesciences, Changzhou, China)

Additional reagents included: Reduced L-glutathione (Solarbio, Beijing, China); DAPI Fluoromount-G mounting medium (SouthernBiotech, 0100-20); Doxycycline (Dox), Formaldehyde, and Sodium butyrate (Sigma-Aldrich); Puromycin, Hygromycin B, and G418 (Invivogen); CellTiter-Glo cell viability assay kit (Promega); Genomic DNA extraction Kit (Tiangen, CDP304, Beijing, China).

### Plasmids

MHV68 ORF50 was subcloned into pEF-FLAG-N or pEF-HA-N using standard molecular biology techniques; pHAGE-OTUD4 WT and the C45A mutant were kindly provided by Dr. Bo Zhong (Wuhan University) [62]; mOTUD4 WT was subcloned into pHAGE-vector and pEF-Myc-N, and the C45A and a DUB deletion mutants (Δ34-155 aa) were generated by site-directed mutagenesis; pCMV-FLAG/Myc-USP7, FLAG-MDM2, and HA-p53 were kindly provided by Dr. Jinfang Zhang (Wuhan University), and USP7 was subcloned into the pEF-HA-N vector [27]; HSV-1 ICP0 was subcloned into pEF-FLAG-N; The TRAF domain of human and murine USP7 (1-208 aa) was subcloned into pGEX-6p-1 (GE Healthcare), and the D164A and W165A mutants (murine are D165A and W166A) were introduced by site-directed mutagenesis; HA-Ub was kindly provided by Dr. Bo Zhong (Wuhan University); Short hairpin RNAs (shRNA) targeting OTUD4 and USP7 were constructed into pLKO.1 (Addgene). The shRNA sequences used in the study: shOTUD4, 5’-TATGCAATGCCTTAGTCATAA-3’; shUSP7, 5’-CAAGCAGTGCTGAAGATAATA-3’; shOTUD4 (m): #1 CCGTGTCACAAGCGCATTTAA, #2 GAGAAATTTGAAGCGTTTATA.

### Peptides

Peptides p1 to p12 (S1A Fig) were synthesized by Sangon Biotech (Shanghai, China) with 95% purity. The Tat-fused peptides in Fig 1C consisted of the corresponding peptides conjugated to the Tat_47-57_ peptide (YGRKKRRQRRR) and two glycine linker residues (QYAOBIO, Suzhou, China). The peptides p8-DRI (nssqsasqnvpprrrqrrkkrGy) and p2-DRI (iesndedavepprrrqrrkkrGy) were synthesized using D-amino acids in a retro-reversed sequence with 95% purity (QYAOBIO, Suzhou, China). The peptide p8-m (YGRKKRRQRRRGGVSQSPSQNSN) was synthesized with 95% purity (QYAOBIO, Suzhou, China). All the peptides were acetylated at the N-terminus and amidated at the C-terminus to mimic the native peptide structure. The peptides were dissolved in PBS to prepare 1 mM stock solutions for *in vitro* experiments and 5 mg/ml stock solutions for *in vivo* experiments.

### Surface plasmon resonance analysis

The interaction between USP7-TRAF and the candidate peptides was assessed by surface plasmon resonance (SPR) using the Biacore T200 system (Cytiva). Recombinant USP7-TRAF protein was immobilized on an activated carboxymethylated dextran (CM5) sensor chip to approximately 8000 response units (RU) using an amine coupling method. Gradient concentrations of peptides were injected at a flow rate of 30 μL/min in running buffer [0.05% (v/v) Tween 20 in PBS]. The results were analyzed using Biacore T200 evaluation software (version 2.0).

### Proximity ligation assay

To detect the in situ interaction between OTUD4 and USP7, a proximity ligation assay (PLA) was conducted using the Duolink kit (DU092101, Sigma-Aldrich) according to the manufacturer’s instructions. Briefly, SLK.BAC-FLAG-OTUD4 cells treated with Dox (1 μg/ml) and the indicated peptides (100 μM) for 24h were fixed with 4% paraformaldehyde for 10 minutes, permeabilized in 0.1% Triton X-100 for 5 minutes, and blocked with 10% goat serum for 1 h. Cells were then incubated overnight with rabbit anti-FLAG (1:500, ABclonal, AE092) and mouse anti-USP7 (1:100, Santa Cruz, sc-137008) monoclonal antibodies. After washing, cells were incubated with secondary antibodies conjugated to PLA probes at 37°C for 1 h. Ligation and amplification were then performed, and slides were mounted with ProLong Gold mounting medium. Images were acquired using a laser scanning confocal microscope (Leica Stellaris 5) and processed using ImageJ [20].

### RNA extraction and RT-qPCR

For assessing the impact of peptides on viral replication, SLK.iBAC-GFP or BCBL-1-Tet-K-RTA cells were treated with doxycycline (Dox, 1 μg/ml) for 24 h in the presence of indicated peptides (100 μM). MLF and NIH3T3 cells were infected with MHV68-GFP at a multiplicity of infection (MOI) of 0.01 in the presence of indicated peptides (100 μM). For assessing the impact of peptides on viral latency, BCBL-1-Tet-K-RTA cells were treated with the indicated peptides (100 μM) for 48 h. Total RNA was extracted using TRIzol reagent (Takara) following the manufacturer’s instructions. cDNA was synthesized using the HiScript II 1st Strand cDNA Synthesis Kit (Vazyme, Nanjing, China). The resulting cDNA was diluted 20-fold and subjected to quantitative PCR (qPCR) analysis using SYBR green qPCR master mix (Bimake, Shanghai, China). The relative quantification of target genes was normalized to ACTB. Primer sequences were listed in Supplementary S1 Table.

### Cell viability assay

SLK.iBAC-GFP cells were seeded in a 96-well plate at a density of 1 × 10^5^ cells/well. The cells were then treated with doxycycline (Dox, 1 μg/ml) and sodium butyrate (0.5 mM) to induce lytic reactivation in the presence of the indicated peptides (100 μM) for 48 h. After treatment and a 30-min equilibration at room temperature, CellTiter-Glo Reagent (Promega, G7570) was added in equal volumes to culture medium. Plates underwent orbital shaking (2 min) for lysis, followed by a 10-min incubation for signal stabilization and luminescence recording (0.25-1 sec integration).

### Protein structure prediction

We leveraged AlphaFold3 with default settings to predict the structural complex of USP7-TRAF and p8, USP7-TRAF and p8m, murine USP7-TRAF and p8, as well as murine USP7-TRAF and p8-m [30]. ChimeraX (1.7.1) was employed to visualize and recolor the structural information.

### GST pull-down assay

For protein purification, GST, GST-USP7-TRAF WT, D164A, or W165A, and GST-USP7- murine TRAF WT, D165A, or W166A were expressed in *E. coli* BL21 (DE3). Protein expression was induced with IPTG (0.5 mM) at 18°C overnight. Following induction, the cells were harvested and lysed in lysis buffer (20 mM Tris-Cl, pH 8.0, 137 mM NaCl, 2.7 mM KCl, 1% Triton X-100, 0.2 mM PMSF), followed by sonication on ice for 15 minutes. The lysates were incubated with glutathione-sepharose beads (Smart-lifesciences, Changzhou, China) at 4°C for 4 h. Bound proteins were extensively washed with wash buffer (20 mM Tris-Cl, pH 8.0, 137 mM NaCl, 2.7 mM KCl, 1% Triton X-100), eluted with elution buffer (20 mM Tris-Cl, pH 8.0, 137 mM NaCl, 2.7 mM KCl, 10 mM reduced L-glutathione), and subsequently dialyzed against dialysis buffer (20 mM Tris-Cl, pH 8.0, 150 mM NaCl, 10% glycerol).

For binding assays, purified GST or GST-USP7-TRAF (human or murine) was incubated with FLAG-OTUD4 (human or murine) expressed in HEK293T cells at 4°C for 4 h. Subsequently, 10 μL of glutathione sepharose was added, followed by rotation for an additional 2 h at 4°C. For competition assays, pGFP, p8, p8m or p8-m peptides (100 μM) were included throughout the incubation process. The beads were extensively washed with lysis buffer (50 mM Tris-Cl, pH 8.0, 150 mM NaCl, 1% Triton X-100, 1 × proteinase inhibitors), and the bound proteins were recovered by boiling with 1 × SDS loading buffer at 95°C for 5 min. The samples were then analyzed by immunoblotting.

### Co-immunoprecipitation

HEK293T cells were transfected with the specified constructs and subsequently treated with the indicated peptide (100 µM) 6 h post-transfection. At 24 h post-treatment, the cells were harvested and lysed in NP-40 lysis buffer (150 mM NaCl, 50 mM Tris-HCl, pH 7.4, 1% NP-40, 1 mM EDTA) containing a protease inhibitor cocktail. Cell lysates were centrifuged at 12,000 rpm for 10 min at 4°C and then the supernatants were incubated with anti-FLAG beads (Dia-An Biotechnology, Wuhan, China) at 4°C for 4 h. The beads were then collected by centrifugation at 4,000 rpm for 2 min at 4°C and washed thoroughly with NP-40 lysis buffer to eliminate nonspecific bound proteins. The binding proteins were released by boiling in SDS sample buffer for 20 min, separated by SDS-PAGE and analyzed by immunoblotting.

### Ubiquitination assay

HEK293T cells were co-transfected with FLAG-m-RTA, HA-Ub, and Myc-mOTUD4 WT, the C45A mutant, or the ΔDUB mutant. After 16 h, the transfected cells were treated with MG132 (10 μM) for an additional 10 h. The cells were collected and lysed in 1% Triton X-100 lysis buffer (150 mM NaCl, 50 mM Tris-HCl, pH 7.4, 1% Triton X-100, 1 mM EDTA, with protease inhibitors) supplemented with 1% SDS and boiled for 5 minutes. The lysates were diluted 10-fold to reduce the SDS concentration to 0.1%. The diluted lysates were subjected to immunoprecipitation with FLAG beads, and the immunoprecipitated proteins were analyzed by immunoblotting.

### Quantification of KSHV infectious units

SLK.iBAC-GFP cells were treated with Dox (1 μg/ml) and sodium butyrate (0.5 mM) to induce lytic reactivation in the presence of indicated peptides (100 μM). The supernatants were collected at the specified time points and used to infect HEK293T cells at appropriate dilutions. The infected cells were collected, fixed, and analyzed by flow cytometry 24 h post-infection. FlowJo 10.0 software was used to analyze the data, and KSHV infectious units were quantified based on the percentage of GFP-positive cells.

### Quantification of viral genome copy number

For assessing the impact of peptides on viral lytic reactivation, BCBL-1-Tet-K-RTA cells were treated with Dox (1 μg/ml) and sodium butyrate (0.5 mM) for 48 h to induce lytic reactivation in the presence of indicated peptides (100 μM). The supernatants (500 μl) were collected and treated with 7.5 U of DNase I (Solarbio, Beijing, China) for 1 h at 37°C. Following DNase I treatment, 30 μl of proteinase K (20 mg/ml, Solarbio, Beijing, China) and 50 μl of 20% SDS were added, and the mixture was incubated at 65°C for 1 h. Genomic DNA was extracted using the phenol-chloroform extraction method [38], resuspended in 50 μl of TE buffer, diluted 20-fold, and quantified by qPCR. A standard curve was generated using serial dilutions of a pEF-FLAG-K-RTA plasmid. For assessing the impact of peptides on viral latency, BCBL-1-Tet-K-RTA cells were treated with the indicated peptides (100 μM) for 48 h. Genomic DNA was extracted using the standard extraction method (Tiangen, Beijing, China). Primer sequences used for quantification were provided in Supplementary S1 Table.

### Generation of stable cell lines

Lentiviruses containing the indicated genes or shRNA constructs were generated as previously described [67]. NIH3T3, MLF, and SLK.iBAC-GFP cells were infected with the lentiviruses, and selected with puromycin (1 μg/ml) 2 days post-infection. For reconstitution experiments, MLF OTUD4 knockout cells were infected with lentiviruses containing vector control, mOTUD4 WT, mOTUD4-C45A, or the DUB depletion mutant, and were selected with puromycin (1 μg/ml). To generate SLK.iBAC-GFP stable cell lines containing the K-RTA mutants, BAC DNA (10 μl, ~2 μg) was transfected into SLK cells seeded in a 6-well plate using Fugene HD (Promega). The transfected cells were selected with hygromycin B (500 μg/ml) at 2 days post-transfection until GFP-positive colonies were sufficiently amplified.

### Hematoxylin and Eosin (H&E) staining

Mouse lung tissue samples were fixed in 10% formalin solution (Sigma) overnight. The fixed tissue samples were then dehydrated, embedded in paraffin, and sectioned into 3-μm slices. The tissue sections were stained using standard H&E staining protocols and analyzed microscopically.

### Statistical analysis

GraphPad Prism (version 8) was used for all statistical analysis. Data are presented as the mean of at least three independent experiments, and error bars denote standard deviation (S.D.). Statistical significance was determined using two-tailed student’s t test or analysis of variance (ANOVA). Significant differences are indicated by p value (*p<0.05, **p<0.01, ***p<0.001).

## Supporting information

S1 Fig Rationally designed peptide p8 demonstrates anti-KSHV activity. (A) A series of peptides containing USP7-binding consensus sequence (P/A/EXXS) derived from the N-terminal region of OTUD4, designated p1 to p12, were highlighted. (B-C) SLK.iBAC-GFP cells were induced with Dox (1 μg/mL) and sodium butyrate (0.5 mM) in the presence of p2 and p8 (100 μM) (B) or the indicated amount of p8 (C). KSHV infectious units were quantified 48 h post-induction. (D) Comparative inhibitory activity of pGFP and p2 against KSHV lytic reactivation in SLK.iBAC-GFP cells. Data are mean ± s.d. of N = 3 independent biological replicates. (E-F) BCBL-1 cells were treated with the indicated peptides (100 μM) for 48 h. The expression of viral latent genes was quantified by qRT-PCR (E), and the relative viral genomic copy number was determined by qPCR (F). Data are mean ± s.d. of N = 3 independent biological replicates.
(TIF)

S2 Fig P8, but not the p8m mutant peptide, represses KSHV lytic reactivation. (A) Bacterially purified GST or GST-USP7-TRAF proteins were incubated with FLAG-OTUD4 expressed in HEK293T cells in the presence of the peptides [pGFP, p8, and p2 (100 μM)] for 4 h. Coomassie blue staining was used to visualize GST-tagged proteins, while western blotting was employed to detect FLAG-OTUD4. Densitometry analysis of the bands was performed using ImageJ. (B-D) HEK293T cells were co-transfected with HA-USP7 and FLAG-MDM2 (B), HA-USP7 and FLAG-ICP0 (C), or HA-p53 and FLAG-USP7 (D). The transfected cells were treated with the indicated peptides (100 µM) 6 h post-transfection. WCLs were collected for immunoprecipitation with anti-FLAG affinity agarose 24 h post-treatment. The input and precipitated samples were analyzed by immunoblotting. Densitometry analysis of the bands was performed using ImageJ. (E-G) SLK.iBAC-GFP cells were induced with Dox (1 µg/mL) and sodium butyrate (0.5 mM) in the presence of the indicated peptides (100 µM). KSHV infectious units were quantified 48 h post-induction (E). Viral gene expression was assessed by RT-qPCR (F), and viral protein levels were detected by immunoblotting (G). Data are mean ± s.d. of N = 3 independent biological replicates (E and F). Densitometry analysis of the bands was performed using ImageJ (G).
(TIF)

S3 Fig P8 inhibits KSHV lytic replication by targeting the RTA-OTUD4-USP7 complex. (A) HEK293T cells were co-transfected with HA-K-RTA and FLAG-OTUD4, and the transfected cells were treated with the indicated peptide (100 µM) 6 h post-transfection. WCLs were collected and analyzed by immunoblotting 24 h post-treatment. Densitometry analysis of the bands was performed using ImageJ. (B) SLK.iBAC-GFP cells were transduced with control shRNA, shRNA targeting OTUD4 or USP7 to generate stable knockdown cell lines, and WCLs were subjected to immunoblotting analysis.
(TIF)

S4 Fig OTUD4 promotes MHV68 lytic replication by enhancing m-RTA deubiquitination and stability, independently of its DUB activity. (A) NIH3T3 cells were transduced with control shRNA or shRNA targeting *Otud4* to generate stable cells, and WCLs were analyzed by immunoblotting. (B) NIH3T3 cells described in S4A Fig were infected with MHV68-GFP (MOI, 0.01). The supernatants containing infectious virions were collected and used to infect BHK21 cells. GFP expression was imaged 48 h post-infection. Scale bars, 100 μm. (C) OTUD4 knockdown NIH3T3 cells described in S4A Fig were infected with MHV68-GFP (MOI, 0.01), and viral titer was determined at the indicated time points post-infection. Data are mean ± s.d. of N = 3 independent biological replicates. (D) OTUD4 knockout MLFs were stably reconstituted with control vector, mOTUD4, mOTUD4-C45A, or mOTUD4-ΔDUB, and WCLs were analyzed by immunoblotting. (E) HEK293T cells were co-transfected with HA-m-RTA and FLAG-mOTUD4, FLAG-mOTUD4-C45A, or FLAG-mOTUD4-ΔDUB, and immunoblotting was performed 24 h post-transfection. Densitometry analysis of the bands was performed using ImageJ. (F) HEK293T cells were co-transfected with HA-m-RTA and different amounts of FLAG-OTUD4/C45A (0, 0.5, 1, or 2 μg). WCLs were collected 24 h post-transfection and analyzed by immunoblotting. Densitometry analysis of the bands was performed using ImageJ. (G) HEK293T cells were co-transfected with FLAG-m-RTA, HA-Ub, and Myc-mOTUD4, C45A, or ΔDUB, and then treated with MG132 (10 μM). Denatured immunoprecipitation with anti-FLAG affinity agarose was performed, followed by immunoblotting.
(TIF)

S5 Fig P8 restricts MHV68 Infection (A) Sequence alignment of human and murine USP7-TRAF and OTUD4 N-terminal domains. Identical residues between species were labeled in red. The positions of p8 were highlighted with a blue background, while divergent amino acids were marked in a plum background. (B) Transparent surface representation of the murine USP7-TRAF domain (plum) bound to peptide p8 (green) predicted by AlphaFold3. (C) Detailed interaction between murine USP7-TRAF (plum) and p8-m (green) based on AlphaFold3. The H-bonds are indicated by dashed lines. (D) Bacterially purified GST or GST fusion proteins (GST-mUSP7-TRAF WT, D165A or W166A) were incubated with FLAG-mOTUD4 expressed in HEK293T cells for 4 h. Coomassie blue staining was used to visualize GST-tagged proteins, while western blotting was employed to detect FLAG-mOTUD4. Densitometry analysis of the bands was performed using ImageJ. (E) Bacterially purified GST or GST-mUSP7-TRAF proteins were incubated with FLAG-mOTUD4 expressed in HEK293T cells in the presence of the peptides [pGFP, p8, and p8-m (100 μM)] for 4 h. Coomassie blue staining was used to visualize GST-tagged proteins, while western blotting was employed to detect FLAG-mOTUD4. Densitometry analysis of the bands was performed using ImageJ. (F-G) NIH3T3 cells were infected with MHV68 (MOI, 0.01) and treated with the indicated peptide (100 µM). Viral titer was determined by plaque assays 48 h post-infection (F), and viral gene expression was measured by RT-qPCR (G). Data are mean ± s.d. of N = 3 independent biological replicates. (H) Surface plasmon resonance (SPR) assay assessing the binding affinity of p8-DRI to USP7-TRAF. Data are mean ± s.d. of N = 3 independent biological replicates. (I) Comparative inhibitory activity of pGFP and p2-DRI (iesndedavepprrrqrrkkrGy) against KSHV lytic reactivation in SLK.iBAC-GFP cells. Data are mean ± s.d. of N = 3 independent biological replicates.
(TIF)

S1 Table Primers used in this study. (PDF)

S2 Table Raw data.
(XLSX)
