# Supplementary figures and images for: Disrupting the OTUD4-USP7 deubiquitinase complex to suppress herpesvirus replication: a novel antiviral strategy
Source: PLoS Pathog. 2025 Apr 10;21(4):e1013052. doi: 10.1371/journal.ppat.1013052 (PMC12047801; doi:10.1371/journal.ppat.1013052)

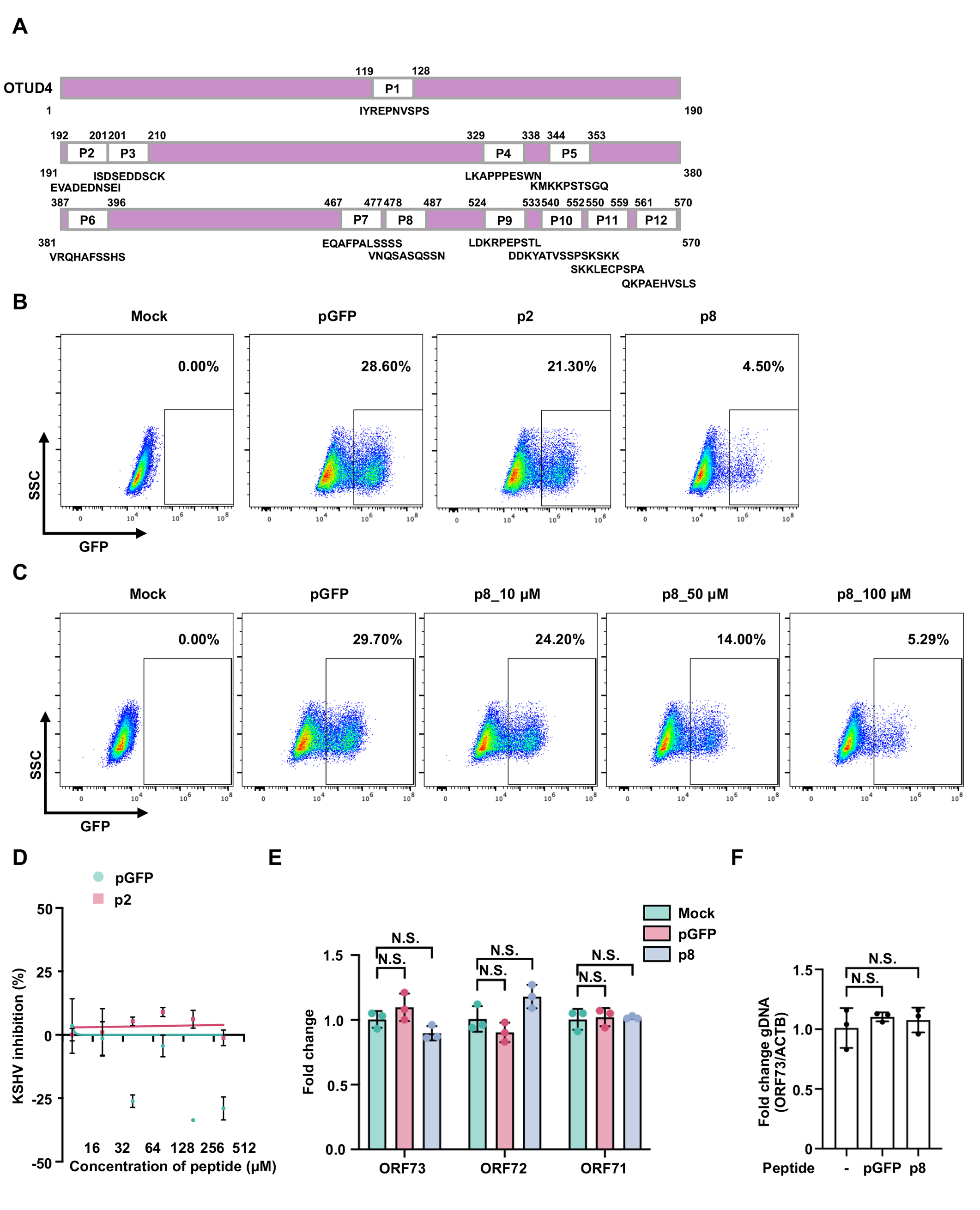

Supplement: S1 Fig — (A) A series of peptides containing USP7-binding consensus sequence (P/A/EXXS) derived from the N-terminal region of OTUD4, designated p1 to p12, were highlighted. (B-C) SLK.iBAC-GFP cells were induced with Dox (1 μg/mL) and sodium butyrate (0.5 mM) in the presence of p2 and p8 (100 μM) (B) or the indicated amount of p8 (C). KSHV infectious units were quantified 48 h post-induction. (D) Comparative inhibitory activity of pGFP and p2 against KSHV lytic reactivation in SLK.iBAC-GFP cells. Data are mean ± s.d. of N = 3 independent biological replicates. (E-F) BCBL-1 cells were treated with the indicated peptides (100 μM) for 48 h. The expression of viral latent genes was quantified by qRT-PCR (E), and the relative viral genomic copy number was determined by qPCR (F). Data are mean ± s.d. of N = 3 independent biological replicates. (TIF) [file ppat.1013052.s001.tif]

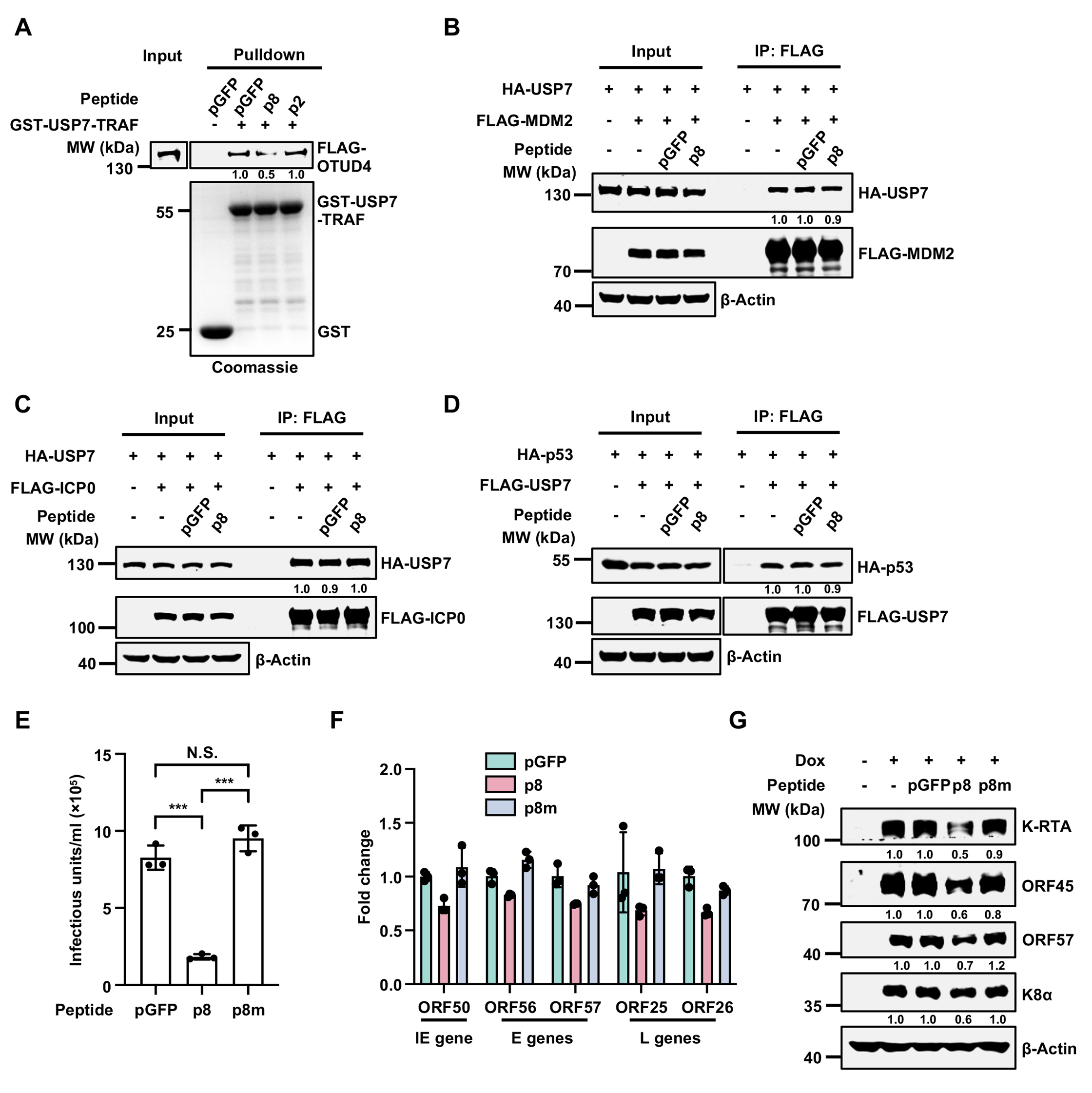

Supplement: S2 Fig — (A) Bacterially purified GST or GST-USP7-TRAF proteins were incubated with FLAG-OTUD4 expressed in HEK293T cells in the presence of the peptides [pGFP, p8, and p2 (100 μM)] for 4 h. Coomassie blue staining was used to visualize GST-tagged proteins, while western blotting was employed to detect FLAG-OTUD4. Densitometry analysis of the bands was performed using ImageJ. (B-D) HEK293T cells were co-transfected with HA-USP7 and FLAG-MDM2 (B), HA-USP7 and FLAG-ICP0 (C), or HA-p53 and FLAG-USP7 (D). The transfected cells were treated with the indicated peptides (100 µM) 6 h post-transfection. WCLs were collected for immunoprecipitation with anti-FLAG affinity agarose 24 h post-treatment. The input and precipitated samples were analyzed by immunoblotting. Densitometry analysis of the bands was performed using ImageJ. (E-G) SLK.iBAC-GFP cells were induced with Dox (1 µg/mL) and sodium butyrate (0.5 mM) in the presence of the indicated peptides (100 µM). KSHV infectious units were quantified 48 h post-induction (E). Viral gene expression was assessed by RT-qPCR (F), and viral protein levels were detected by immunoblotting (G). Data are mean ± s.d. of N = 3 independent biological replicates (E and F). Densitometry analysis of the bands was performed using ImageJ (G). (TIF) [file ppat.1013052.s002.tif]

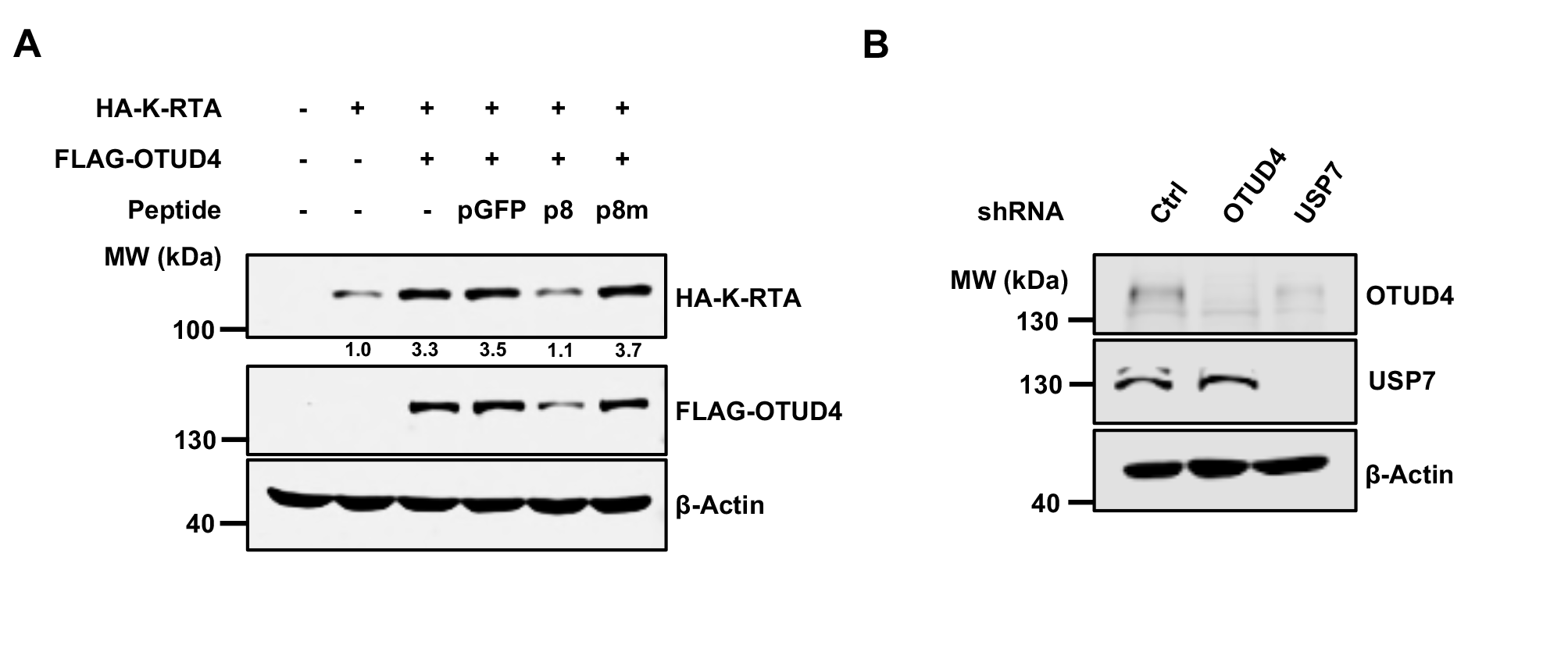

Supplement: S3 Fig — (A) HEK293T cells were co-transfected with HA-K-RTA and FLAG-OTUD4, and the transfected cells were treated with the indicated peptide (100 µM) 6 h post-transfection. WCLs were collected and analyzed by immunoblotting 24 h post-treatment. Densitometry analysis of the bands was performed using ImageJ. (B) SLK.iBAC-GFP cells were transduced with control shRNA, shRNA targeting OTUD4 or USP7 to generate stable knockdown cell lines, and WCLs were subjected to immunoblotting analysis. (TIF) [file ppat.1013052.s003.tif]

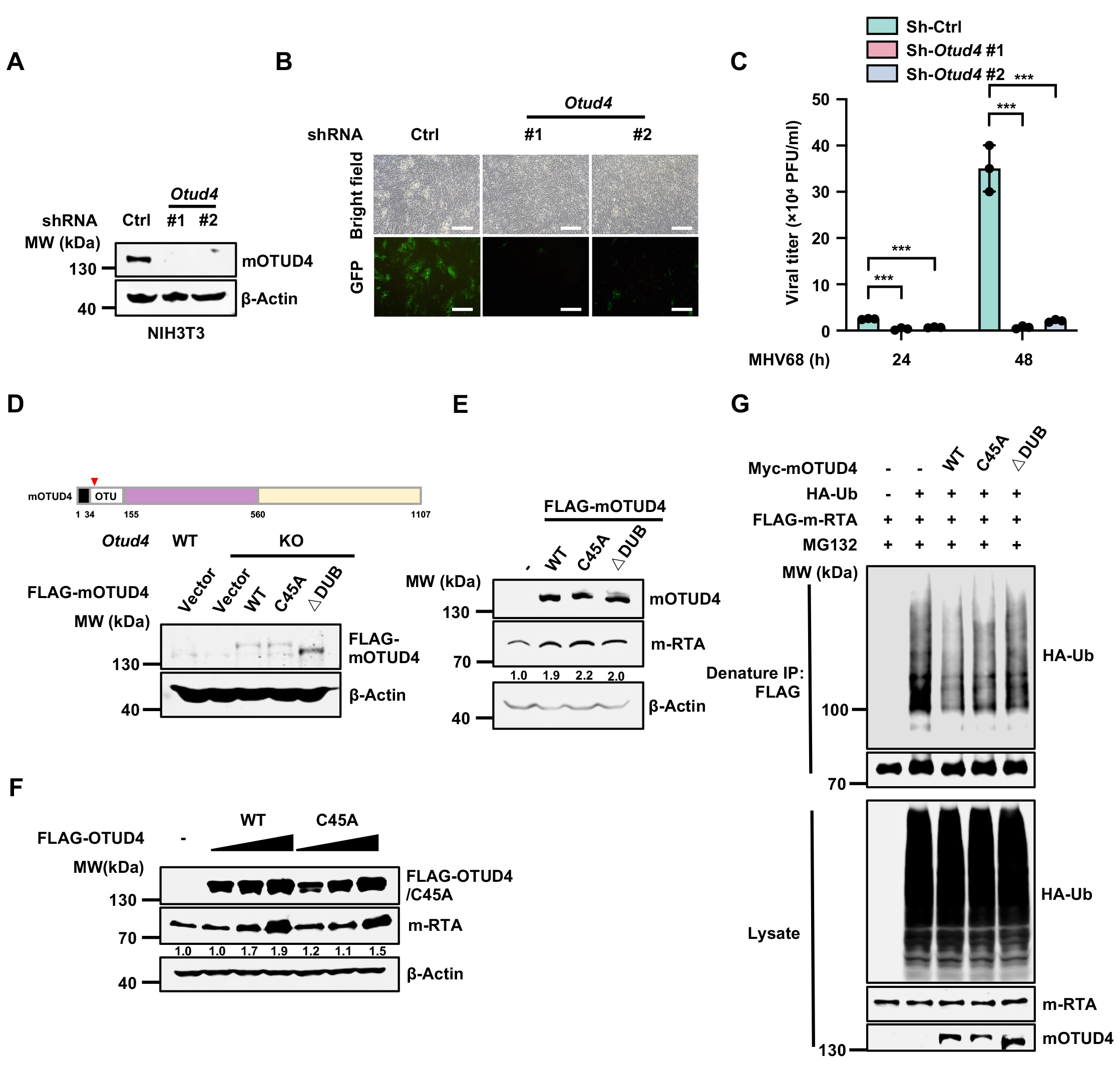

Supplement: S4 Fig — (A) NIH3T3 cells were transduced with control shRNA or shRNA targeting Otud4 to generate stable cells, and WCLs were analyzed by immunoblotting. (B) NIH3T3 cells described in S4A Fig were infected with MHV68-GFP (MOI, 0.01). The supernatants containing infectious virions were collected and used to infect BHK21 cells. GFP expression was imaged 48 h post-infection. Scale bars, 100 μm. (C) OTUD4 knockdown NIH3T3 cells described in S4A Fig were infected with MHV68-GFP (MOI, 0.01), and viral titer was determined at the indicated time points post-infection. Data are mean ± s.d. of N = 3 independent biological replicates. (D) OTUD4 knockout MLFs were stably reconstituted with control vector, mOTUD4, mOTUD4-C45A, or mOTUD4-ΔDUB, and WCLs were analyzed by immunoblotting. (E) HEK293T cells were co-transfected with HA-m-RTA and FLAG-mOTUD4, FLAG-mOTUD4-C45A, or FLAG-mOTUD4-ΔDUB, and immunoblotting was performed 24 h post-transfection. Densitometry analysis of the bands was performed using ImageJ. (F) HEK293T cells were co-transfected with HA-m-RTA and different amounts of FLAG-OTUD4/C45A (0, 0.5, 1, or 2 μg). WCLs were collected 24 h post-transfection and analyzed by immunoblotting. Densitometry analysis of the bands was performed using ImageJ. (G) HEK293T cells were co-transfected with FLAG-m-RTA, HA-Ub, and Myc-mOTUD4, C45A, or ΔDUB, and then treated with MG132 (10 μM). Denatured immunoprecipitation with anti-FLAG affinity agarose was performed, followed by immunoblotting. (TIF) [file ppat.1013052.s004.tif]

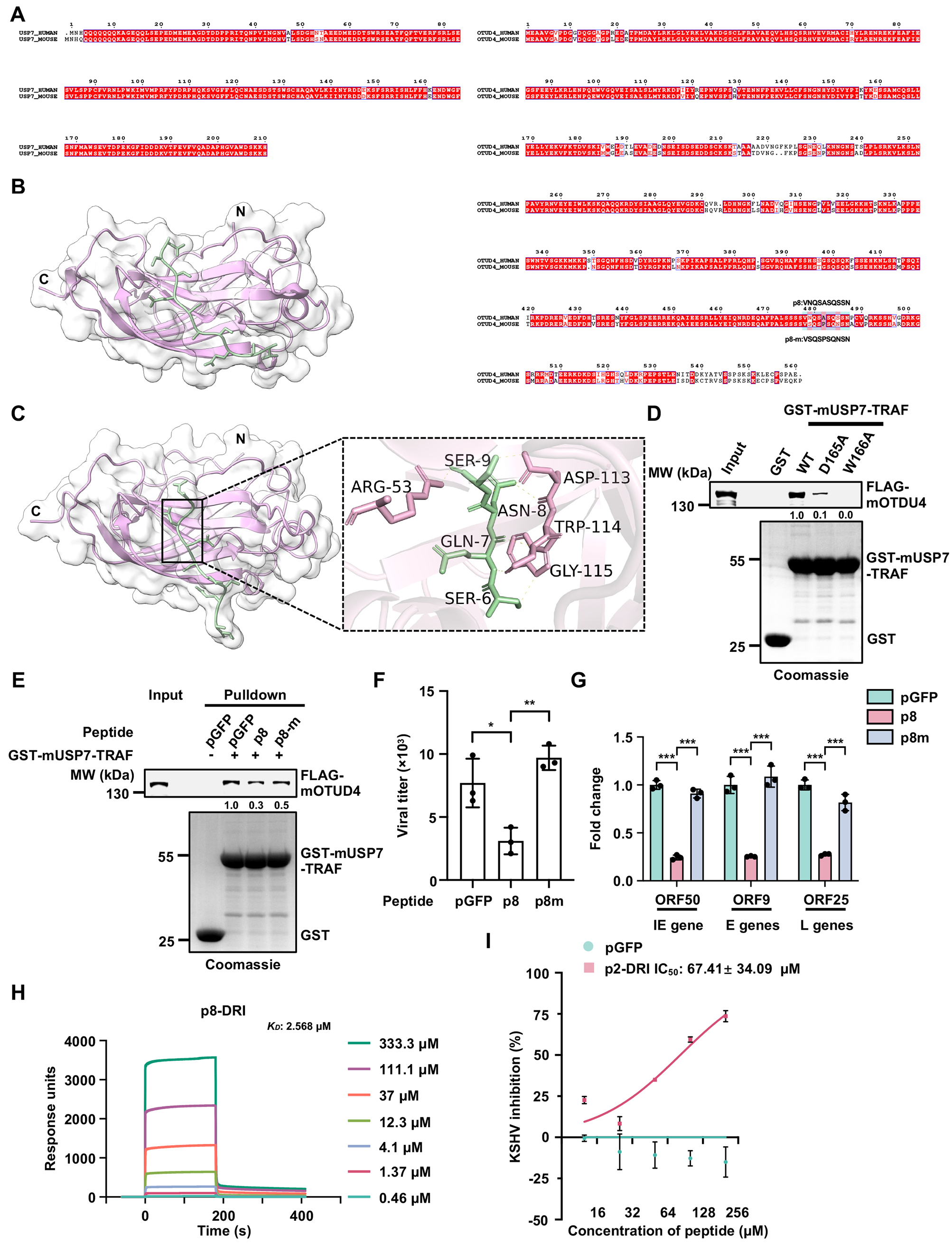

Supplement: S5 Fig — (A) Sequence alignment of human and murine USP7-TRAF and OTUD4 N-terminal domains. Identical residues between species were labeled in red. The positions of p8 were highlighted with a blue background, while divergent amino acids were marked in a plum background. (B) Transparent surface representation of the murine USP7-TRAF domain (plum) bound to peptide p8 (green) predicted by AlphaFold3. (C) Detailed interaction between murine USP7-TRAF (plum) and p8-m (green) based on AlphaFold3. The H-bonds are indicated by dashed lines. (D) Bacterially purified GST or GST fusion proteins (GST-mUSP7-TRAF WT, D165A or W166A) were incubated with FLAG-mOTUD4 expressed in HEK293T cells for 4 h. Coomassie blue staining was used to visualize GST-tagged proteins, while western blotting was employed to detect FLAG-mOTUD4. Densitometry analysis of the bands was performed using ImageJ. (E) Bacterially purified GST or GST-mUSP7-TRAF proteins were incubated with FLAG-mOTUD4 expressed in HEK293T cells in the presence of the peptides [pGFP, p8, and p8-m (100 μM)] for 4 h. Coomassie blue staining was used to visualize GST-tagged proteins, while western blotting was employed to detect FLAG-mOTUD4. Densitometry analysis of the bands was performed using ImageJ. (F-G) NIH3T3 cells were infected with MHV68 (MOI, 0.01) and treated with the indicated peptide (100 µM). Viral titer was determined by plaque assays 48 h post-infection (F), and viral gene expression was measured by RT-qPCR (G). Data are mean ± s.d. of N = 3 independent biological replicates. (H) Surface plasmon resonance (SPR) assay assessing the binding affinity of p8-DRI to USP7-TRAF. Data are mean ± s.d. of N = 3 independent biological replicates. (I) Comparative inhibitory activity of pGFP and p2-DRI (iesndedavepprrrqrrkkrGy) against KSHV lytic reactivation in SLK.iBAC-GFP cells. Data are mean ± s.d. of N = 3 independent biological replicates. (TIF) [file ppat.1013052.s005.tif]
