# Supplementary material for: Disrupting the OTUD4-USP7 deubiquitinase complex to suppress herpesvirus replication: a novel antiviral strategy
Source: PLoS Pathog. 2025 Apr 10;21(4):e1013052. doi: 10.1371/journal.ppat.1013052 (PMC12047801; doi:10.1371/journal.ppat.1013052)
Supplement: S1 Table — (PDF) [file ppat.1013052.s006.pdf]

Supplementary Table 1 primers used in this study

Gene	Sequence	
KSHV ORF50	CGCAATGCGTTACGTTGTTG	GCCCGGACTGTTGAATCG
KSHV ORF56	CACAGATTCCCGTCAATACAAA	GTATCTTCAGTAGGCGGCAGAG
KSHV ORF57	CATCCTAGAGGACTCTGT	TTGCTCGTCTTCCAGTGT
KSHV ORF25	ACAGTTTATGGCACGCATAGTG	GGTTCTCTGAATCTCGTCGTGT
KSHV ORF26	GCTCGAATCCAACGGATTTG	AATAGCGTGCCCCAGTTGC
KSHV ORF73	CCTGGAAGTCCCACAGTGTT	AGACACAGGATGGGATGGAG
KSHV ORF72	TCTTGAAATTGAGCCGCGCT	CAACGCGACCACGTTAGGTT
KSHV ORF71	TAATTGGAGCTCTTAGAGC	ACGCGGGTCTAAGTGAAGCA
MHV68 ORF50	AGAAACCCACAGCTCGCACTT	CAATATGCTGGACAGGCGTATC
MHV68 ORF9	TGCATGCAAGTTTGTCCAGTCT	CTTCCCCCAGTTACTCATTGTTTG
MHV68 ORF25	ACGTGCCAAGTGAATTCATG	TTCATGTGCATGGCCAACAT
Mouse Atcb	TCTACGAGGGCTATGCTCTCC	TCTTTGATGTCACGCACGATTTC
Human ATCB	GTTGTCGACGACGAGCG	GCACAGAGCCTCGCCTT
